# Effect size quantification for interrupted time series analysis: implementation in R and analysis for Covid-19 research

**DOI:** 10.1186/s12982-022-00118-7

**Published:** 2022-11-11

**Authors:** Yael Travis-Lumer, Yair Goldberg, Stephen Z. Levine

**Affiliations:** 1 Faculty of Industrial Engineering and Management, Israel Institute of Technology, 3200003 Haifa, Israel; 2 School of Public Health, University of Haifa, Haifa, Israel

**Keywords:** Interrupted time series, Effect size, Relative risk, Cohen’s *d*, Mortality, Covid-19

## Abstract

**Background:**

Interrupted time series (ITS) analysis is a time series regression model that aims to evaluate the effect of an intervention on an outcome of interest. ITS analysis is a quasi-experimental study design instrumental in situations where natural experiments occur, gaining popularity, particularly due to the Covid-19 pandemic. However, challenges, including the lack of a control group, have impeded the quantification of the effect size in ITS. The current paper proposes a method and develops a user-friendly R package to quantify the effect size of an ITS regression model for continuous and count outcomes, with or without seasonal adjustment.

**Results:**

The effect size presented in this work, together with its corresponding 95% confidence interval (CI) and P-value, is based on the ITS model-based fitted values and the predicted counterfactual (the exposed period had the intervention not occurred) values. A user-friendly R package to fit an ITS and estimate the effect size was developed and accompanies this paper. To illustrate, we implemented a nation population-based ITS study from January 2001 to May 2021 covering the all-cause mortality of Israel (n = 9,350 thousand) to quantify the effect size of Covid-19 exposure on mortality rates. In the period unexposed to the Covid-19 pandemic, the mortality rate decreased over time and was expected to continue decreasing had Covid-19 not occurred. In contrast, the period exposed to the Covid-19 pandemic was associated with an increased all-cause mortality rate (relative risk = 1.11, 95% CI = 1.04, 1.18, P < 0.001).

**Conclusion:**

For the first time, the effect size in ITS: was quantified, can be estimated by end-users with an R package we developed, and was demonstrated with data showing an increase in mortality following the Covid-19 pandemic. ITS effect size reporting can assist public health policy makers in assessing the magnitude of the entire intervention effect using a single, readily understood measure.

**Supplementary Information:**

The online version contains supplementary material available at 10.1186/s12982-022-00118-7.

## Background

Interrupted time series (ITS) analysis is a time series regression model that aims to evaluate the effect of an intervention or treatment that has been implemented beginning at a well-defined starting point in time. An ITS study design is a quasi-experimental study design instrumental in situations where natural experiments occur, such as when the government imposes Covid-19 attenuation strategies to reduce Covid-19 infection and death. Due to the Covid-19 pandemic, ITS analysis has gained popularity as a study design to evaluate the effect of Covid-19 attenuation strategies (e.g., lockdowns) on Covid-19 infections, and on economic, psychiatric, and psychological outcomes [1–4]. Despite available tutorials and books on ITS [5–8], it is unclear how to quantify the effect size in ITS. This is particularly relevant today because an effect size estimate for ITS will allow us to evaluate the overall effect of the Covid-19 intervention which is more readily interpretable than changes in regression coefficients. We aim to demonstrate how to quantify the effect size based on ITS analysis, provide an R package to quantify the effect size in ITS, and present an example using national population-based all-cause mortality data.

A primary challenge of an ITS is the absence of a control group since the entire study population is exposed to the same intervention simultaneously, and this introduces complexity in quantifying the effect size. If, however, the time series covers adequate observations from before and after the intervention, one can compare the observations from the intervention period (exposed to the intervention or during the Covid-19 pandemic) to the observations from the pre-intervention period (unexposed to the intervention). Nonetheless, simple before and after comparisons, such as the mean difference between the exposed and unexposed periods, are inappropriate as this scenario compares two disjoint segments of a time series, potentially with the added complexity of a time trend. Hence, the common practice is to fit a regression model to account for the pre-intervention time trend and the post-intervention level and slope change (where the level describes the initial value, and the slope describes the average trend over time).

While the post-intervention level and slope change are reported widely in ITS analysis, it is unclear how to quantify the overall effect size, especially when the outcome is not continuous. For example, with logistic and Poisson regression models, it is often easier to understand and convey concepts like relative risk (RR) to the public and policymakers instead of directly interpreting the meaning of the regression coefficients.

Effect size reporting is desirable [9]; however, it is unclear if and how one can define and estimate such effect sizes in ITS, in the absence of a control group. Preliminary steps to address this lacuna have been undertaken, but only in restricted scenarios. Previously, the RR of ITS has been reported for Poisson regression models with no post-intervention slope change [5–7]. Several reviews of ITS studies summarized that estimates of intervention effects are mostly reported as the change in regression coefficients [10, 11].

### Interrupted time series analysis

**Fig. 1:**
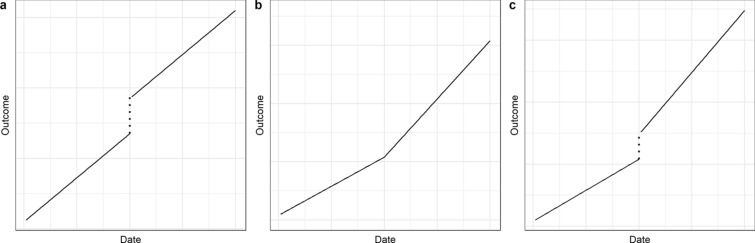
Examples of impact models used in interrupted time series analysis: **a** level change; **b** slope change; **c** level and slope change

An ITS regression model for a continuous outcome can be described as follows. Assume that we observe a time series of continuous outcomes, denoted by ${Y}_{t}$, for times $1\le t\le T$. Assume that some intervention occurred starting from some time ${t}^{*}>1$ and until $T$. Let ${X}_{t}={1}_{\{t\ge {t}^{*}\}}$ denote a binary indicator indicating whether the intervention occurred at time $t$, or not. An ITS linear regression model corresponding to both a level change and a slope change following the intervention, can be formalized as follows:

1 $$E\left({Y}_{t}\mid \hspace{0.25em}{X}_{t},t\right)={\beta }_{0}+{\beta }_{1}\cdot t+{\beta }_{2}\cdot {X}_{t}+{\beta }_{3}\cdot (t-{t}^{*}){X}_{t},$$

where ${\beta }_{0}$ is the pre-intervention initial level, ${\beta }_{1}$ is the time trend coefficient, ${\beta }_{2}$ is the post-intervention level change, and ${\beta }_{3}$ is the post-intervention slope change. The model-based counterfactual values can be obtained from model (1) with ${X}_{t}$ set to zero. In some cases, it may be appropriate to assume an ITS model with no slope change (i.e. ${\beta }_{3}=0$) or with no level change (i.e. ${\beta }_{2}=0$). The choice of appropriate model depends on the hypothesized intervention effect, also known as the impact model. Specifically, the impact model expresses our prior assumption regarding how the intervention will affect the outcome of interest. In particular, a decision is required as to whether the change following the intervention will be gradual, include a level change, and follow the intervention immediately or after a delay (termed a lag). Figure 1 presents three different impact model scenarios. These scenarios can be generalized to include also short-term effects or delayed effects. Finally, the regression model (1) can be generalized to include different types of outcomes (using a generalized linear model), accommodate a lagged intervention effect, and control for seasonality and non-linear time trends (using seasonal terms and flexible spline functions) [5].

The current paper aims to propose a method to quantify the effect size of a general ITS regression model for continuous and count outcomes, with or without seasonal adjustment. We propose to quantify the effect size by comparing the model-based fitted values for the intervention period with their model-based counterfactual values. The counterfactual values are the model predictions had the intervention not occurred, and so continue the existing trend based on the unexposed period. Two possible ways to quantify the effect size are presented, depending on whether the outcome is based on continuous data, in which case we use Cohen’s *d* as the effect size, or count data, in which case we use the RR. Based on this method, we develop a user-friendly R package. We provide a tutorial to use this package, demonstrate ITS analysis for both outcomes, and estimate the effect size together with its corresponding 95% CI and P-value. We demonstrate the use of the method and package to quantify the effect size of the association between the Covid-19 pandemic and all-cause mortality rates based on national population-based data.

## Results

### Scenarios to quantify the effect size in ITS

This manuscript proposes to quantify the effect size by comparing the model-based fitted values for the intervention period with their model-based counterfactual values. We first discuss the case of continuous outcomes, and then discuss the case of count outcomes. In both cases, we focus on quantifying the effect size, where for continuous outcomes we are averaging standardized differences, and for count outcomes we are averaging risk ratios.

### Effect size for continuous outcomes in ITS: Cohen’s d

For continuous outcomes, the effect size is defined by Cohen’s *d*, where Cohen’s *d* is calculated by dividing the overall mean difference for the intervention period with the pooled standard deviation [12]. To obtain the mean difference for each time point during the intervention period, we subtract the model prediction had the intervention not occurred (the model-based counterfactual value) from the model-based fitted values. Next, we average the differences to obtain the overall mean difference for the entire intervention period. Finally, we divide the overall mean difference with the pooled standard deviation of the predictions. In Additional file 1: Appendix A of the online supplemental, we show that the standardized mean difference is $\widehat{d}=\frac{\widehat{{\beta }_{2}}+\widehat{{\beta }_{3}}\cdot \left(\frac{T-{t}^{*}}{2}\right)}{{S}_{p}},$ where $\widehat{{\beta }_{2}}$ and $\widehat{{\beta }_{3}}$ are the estimated regression coefficients of model (1), ${S}_{p}$ is the pooled standard deviation defined by ${S}_{p}=\sqrt{\frac{{S}_{1}^{2}+{S}_{2}^{2}}{2},}$ and where ${S}_{1}^{2}$ and ${S}_{2}^{2}$ are the estimated variances of the fitted values and the predicted counterfactual values, respectively. For completeness, ${S}_{1}^{2}$ and ${S}_{2}^{2}$ are formally defined in Additional file 1: Appendix A.

The corresponding 95% confidence interval and *P*-value can be obtained by parametric bootstrap implemented in our R package (see Additional file 1: Appendix A).

### Effect size for count outcomes in ITS: the relative risk

Assume now that we observe a time series of count outcomes which we model using a Poisson regression model. Then the effect size is defined by the mean RR for the intervention period. Specifically, for each time-point during the intervention period, we divide the model-based fitted value with its model-based counterfactual value to obtain the pointwise RR. We then average these RRs and obtain that the mean relative risk can be shown (Additional file 1: Appendix A) to be equal to

$${\text{RR}}={\text{exp}}\left(\widehat{{\beta }_{2}}+\widehat{{\beta }_{3}}\cdot \left(\frac{T-{t}^{*}}{2}\right)\right),$$

where $\widehat{{\beta }_{2}}$ and $\widehat{{\beta }_{3}}$ are the estimated regression coefficients of the Poisson regression model (detailed in Additional file 1: Appendix A). Denote by $\widehat{MD}=\widehat{{\beta }_{2}}+\widehat{{\beta }_{3}}\cdot \left(\frac{T-{t}^{*}}{2}\right)$ the expression inside the exponent. Then the 95% confidence interval (CI) corresponding to the RR is ${\text{CI}}=\left({\text{exp}}\left(\widehat{MD}-1.96\cdot {\widehat{\sigma }}_{MD}\right), {\text{exp}}\left(\widehat{MD}+1.96\cdot {\widehat{\sigma }}_{MD}\right)\right)$, and the corresponding *P*-value is *P* $=2\left(1-\Phi \left(\frac{\widehat{MD}}{{\widehat{\sigma }}_{MD}}\right)\right)$, where 1.96 is the 97.5% percentile point of the standard normal distribution, $\Phi \left(\cdot \right)$ is the cumulative distribution function of a standard normal random variable, and where ${\widehat{\sigma }}_{MD}$ is defined in Additional file 1: Appendix A.

### R package

The R package `its2es’ is available at https://github.com/Yael-Travis-Lumer/its2es. This package aims to provide user-friendly functions to fit an ITS regression model and to quantify the effect size. This is implemented for continuous and count outcomes, with and without seasonal adjustments. There are several methods to control for seasonal patterns. Here we use the commonly chosen Fourier terms, that consist of pairs of sine and cosine functions of different frequencies [5].

The its2es R package includes two main functions; one function for continuous outcomes and one function for count outcomes; both functions can adjust for seasonality. The package also includes the dataset analyzed in the data analysis section. The function its_lm() fits model (1) to continuous outcomes. The function in its basic form reads as follows:

its_lm(data, form, time_name, intervention_start_ind, freq, seasonality, impact_model, counterfactual).

The eight arguments are described in Table 1. The regression model (1) can be generalized to include also additional covariates, such as seasonal terms and splines. This is also implemented in our its2es R package, that allows the user to define the regression formula and the corresponding covariates for the analysis using the form argument (Table 1).

**Table 1:** Description of the arguments in the function its_lm()

Argument name	Description
Data	The data frame corresponding to the supplied formula, existing of at least 2 variables: (1) the outcome, and (2) a vector of time points
Form	A formula with the response on the left, followed by the ~ operator, and the covariates on the right, separated by + operators. The formula should not contain an offset term
Time_name	A string giving the name of the time variable. The time variable may or may not be supplied as a covariate in the formula
Intervention_start_ind	Numeric—a number between 1 and nrow(data)-1 stating the time point of the start of the intervention
Freq	A positive integer describing the frequency of the time series
Seasonality	A string specifying whether seasonality should be considered. Possible options include “none” corresponding to no seasonal adjustment, “full” corresponding to using freq-1 Fourier terms to model the seasonal component, and “significant” indicating whether only the significant Fourier terms should be considered in the seasonal adjustment. Default value is “none”
Impact_model	A string specifying the assumed impact model. Possible options include “full” corresponding to a model including both a level change and a slope change, “level” corresponding to a model including just a level change, and “slope” corresponding to a model including just a slope change. Default value is “full”
Counterfactual	Logical—indicating whether the model-based counterfactual values should also be returned as an additional column in the data. Default value is FALSE, in which case the counterfactual values are not returned

The function returns a list with three elements: (i) the fitted regression model, (ii) the model summary, as a list, including also the estimated mean difference and Cohen’s *d*, together with the corresponding 95% CI and *P*-value, and (iii) the original data together with the model-based fitted values (and possibly also the model-based counterfactual values, depending on the user’s choice).

The function its_poisson() fits a Poisson regression model to count outcomes. This function includes two additional arguments relevant only to Poisson regression: offset_name—the name of the offset term (if it exists) and over_dispersion—a logical indicating whether the data is over-dispersed (when the variance is greater than the mean), in which case a quasi-Poisson model is used instead. Like the its_lm() implementation above, the function returns a list with the same three elements, except that Cohen’s *d* is replaced by the RR.

The its2es R package contains a README file and a tutorial explaining how to load the data used in the data analysis section, fit ITS regression models to the data, obtain the relevant effect sizes, and plot the model predictions. The tutorial is available with the R package from https://github.com/Yael-Travis-Lumer/its2es.

### Data analysis example

Here we use an ITS design to quantify the effect of exposure to the Covid-19 pandemic on monthly all-cause mortality rates in Israel. The monthly number of deaths and the yearly population size were reported by the Israel CBS (Central Bureau of Statistics) [13] for males and females of different age groups. We used interpolation to estimate the monthly population size from the yearly data. Hence, the joined data consists of the estimated monthly population size, and the monthly number of deaths, for the period between January 2001 and May 2021. The data is available with this paper as part of the its2es R package.

### Covariates

The interval from January 2001 to February 2020 was classified as the pre-Covid-19 pandemic unexposed period. The first confirmed case of Covid-19 in Israel was on 27 February 2020, and the first lockdown started on 14 March 2020. Hence, we classified the period exposed to the Covid-19 pandemic as starting from March 2020 and until the end of the study on May 2021. The study covariates in our analysis were time (a monthly sequence from January 2001 to May 2021), exposure to the Covid-19 pandemic (classified as unexposed to the Covid-19 pandemic before March 2020 coded 0, and exposed from March 2020 to May 2021 coded 1), and the interaction between time and exposure to the Covid-19 pandemic period. Additional covariates include seasonal Fourier terms to model the seasonal factors, and an offset term (log of the monthly population size) to model event rates.

### Analytic plan

To quantify the effect of Covid-19 pandemic on monthly all-cause mortality rates we modelled the monthly number of deaths (count) using a Poisson regression model. The Poisson regression model included the covariates time, exposure to the Covid-19 period, the interaction between the two, an offset term, and seasonal Fourier terms.

The robustness of the primary Poisson regression model was challenged in a series of seven sensitivity analyses addressing groups with different demographic characteristics. We conducted seven separate sensitivity analyses to examine effect size modification by sex and age differences known to influence mortality rates [14], and specifically by: sex across all age-groups, sex for persons aged over 60, and for all persons aged below 20, above 20 and below 60, and over 60.

### Implementation in R

The implementation of this data analysis in R is detailed in Additional file 1: Appendix B of the online supplemental material.

### Data analysis results

**Fig. 2:**
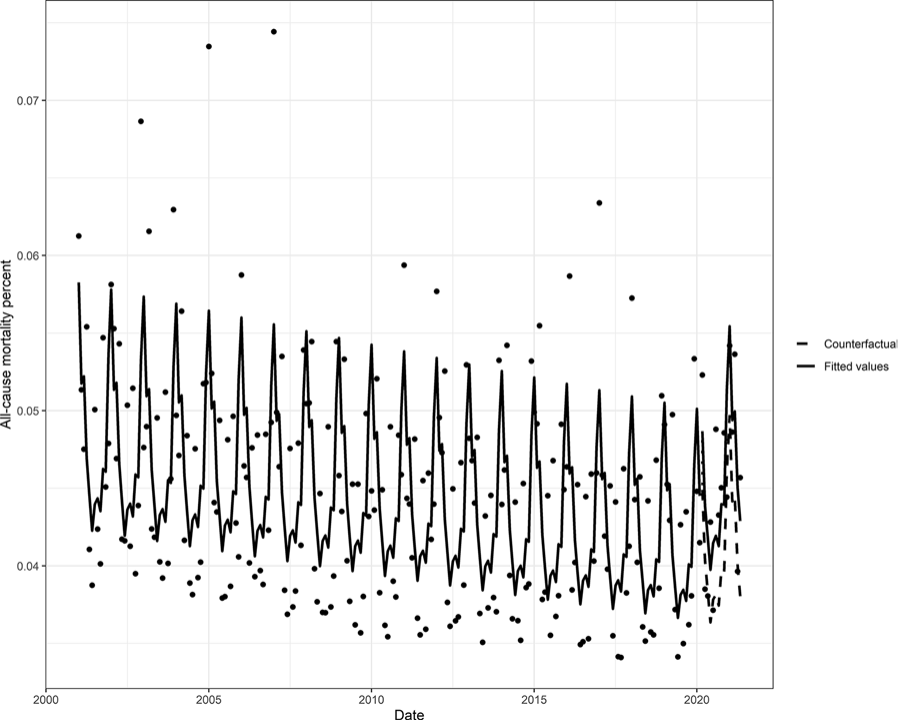
The monthly all-cause mortality percent modeled using a Poisson regression with an offset, and seasonal adjustments. The counterfactual refers to the predicted values had no Covid-19 occurred, and the fitted values are estimated based on the regression mode

In the period unexposed to the Covid-19 pandemic (1 January 2001 to 1 February 2020), the all-cause mortality rate decreased over time and, as observed by the counterfactual, was expected to continue decreasing had the Covid-19 pandemic not occurred (Fig. 2). In contrast to the counterfactual, the period exposed to the Covid-19 pandemic (1 March 2020 to 1 May 2021) was associated with an increased all-cause mortality rate, displaying a disparity between the model predictions and the expected counterfactual values (Fig. 2).

The disparity between the model predictions and the expected counterfactual values is quantified by the effect size, where the effect size for count outcomes is measured by the RR. The exposed Covid-19 pandemic period showed a statistically significant (P < 0.05) increase in the RR of the number of deaths (RR = 1.11, 95% CI = 1.04, 1.18) compared to the counterfactual. That is, there was a statistically significant excess mortality of about 11%.

### Sensitivity analyses

Sensitivity analyses were undertaken to consider groups with potentially differential mortality risks based on their demographic characteristics (Table 2). The results of the primary analysis replicated in a series of sensitivity analyses restricted to groups of males and females across all ages (Additional file 1: Figure S1), males and females aged over 60 (Additional file 1: Figure S2), and among persons aged between 20 and 60, and over 60 (Additional file 1: Figure S3). The Covid-19 pandemic had a null effect on the RR of mortality among children and persons aged below 20 (Table 2). As shorter pre-intervention intervals could have confounded the analysis, we also repeated the primary analysis using only part of the mortality data, keeping observations from January 2015 and onwards. We obtained a very similar effect size (Table 2, Additional file 1: Figure S4), which demonstrates that the effect size is robust and remains stable even when only using 5 years of historical data (instead of 20).

**Table 2:** Comparison of the Covid-19 regression coefficients, together with the RR, for all Poisson regression models

Model	Covid-19 level change (95% CI)	P value	Covid-19 slope change (95% CI)	P value	Covid-19 RR (95% CI)	P value
Primary poisson	0.08 (−0.03, 0.19)	0.15	0.00 (−0.01, 0.02)	0.66	1.11 (1.04, 1.18)	P < 0.001
Males	0.11 (0.00, 0.21)	0.06	0.00 (−0.01, 0.02)	0.65	1.13 (1.07, 1.21)	P < 0.001
Females	0.05 (−0.06, 0.17)	0.35	0.00 (−0.01, 0.02)	0.68	1.08 (1.01, 1.15)	0.03
Males over 60	0.14 (0.03, 0.25)	0.01	0.00 (−0.01, 0.01)	0.84	1.16 (1.09, 1.23)	P < 0.001
Females over 60	0.07 (−0.05, 0.18)	0.26	0.00 (−0.01, 0.02)	0.72	1.09 (1.02, 1.16)	0.01
Aged 0–19	−0.32 (−0.56, −0.10)	0.01	0.04 (0.01, 0.06)	0.01	0.92 (0.82, 1.04)	0.2
Aged 20–59	0.04 (−0.09, 0.17)	0.52	0.01 (−0.01, 0.02)	0.41	1.09 (1.01, 1.17)	0.02
Aged over 60	0.10 (−0.01, 0.21)	0.07	0.00 (−0.01, 0.01)	0.78	1.12 (1.05, 1.19)	P < 0.001
Short pre-intervention interval	0.09 (−0.04, 0.22)	0.19	0.00 (−0.01, 0.02)	0.67	1.12 (1.02,1.22)	0.02

## Discussion

The current study has demonstrated how to quantify the effect size of an intervention on outcomes using an ITS study design. Specifically, we considered the case of both continuous and count outcomes that are the two most common outcomes in ITS analysis, with or without adjustment for seasonality. The current study is the first to offer a method and implementation to report effect sizes in ITS. We illustrated this method and implementation of the ITS regression model to compute the model-based fitted values and the predicted counterfactual values and quantified the effect size of RR for all-cause mortality due to Covid-19.

Our methodology is based on the concept of comparing the ITS model-fitted values to the predicted counterfactual values, thus enabling effect size estimation. The effect size, together with its CI and P-value, is based on the post-intervention level and slope changes, thereby extending the previous approach of reporting the regression coefficients. The R package, its2es, which includes easy-to-use functions that fit an ITS, and estimate the effect size, is available with this work. This package enables effect size estimation for both continuous and count outcomes, with or without seasonal adjustments.

We demonstrated our methodological approach on real-world time series data on all-cause mortality rates based on national population-based data from Israel spanning 20 years, including the Covid-19 pandemic exposed period. We found that the period exposed to the Covid-19 pandemic was associated with a statistically significant increase in the all-cause mortality rates in Israel. Generally, this result replicated restricting to different demographic groups and a shorter pre-intervention interval of five rather than 20 years. Additionally, these results are consistent with previous studies [15, 16] which also found an increase of about 10–12% in all-cause mortality rates in Israel during the Covid-19 period, even though both used a different statistical model and other study covariates. That is, the results are robust to the chosen statistical model. The exact mechanism for the change in the all-cause mortality rate during the Covid-19 period is unknown. It may have changed owing to the virus, and/or other medical conditions that went untreated due to fear of going to a hospital. Here we try to capture all of the excess mortality by one measure only (the Covid-19 period); clearly, attributing the excess mortality to each possible cause is more complicated and is beyond the scope of this paper and data. Finally, note that the decrease in the all-cause mortality rate observed during the period unexposed to the Covid-19 pandemic can be explained by the increase in life expectancy in Israel, and by the growing number of younger families.

### Limitations

First note that an ITS regression model assumes a linear relationship between the outcome and the time covariate (with possible seasonal fluctuations). However, in some cases, the relationship between the time covariate and the outcome may be non-linear, in which case flexible spline functions may be added to the regression model as additional covariates [5]. This can also be implemented in the its2es R package (see Additional file 1: Appendix B of the online supplemental material). An additional assumption of standard regression models is that the observations are independent. However, time series data is usually highly correlated. Fortunately, this correlation is usually explained by seasonality, which can be adjusted for in the ITS regression model. This assumption can be verified using residual autocorrelation and partial autocorrelation plots.

Our approach to quantifying effect sizes in ITS uses the model-based predicted values and not the actual values. This is because it is impossible to observe the true counterfactual values in an ITS study design, as we cannot observe what might have been had the intervention not occurred. Hence, our effect size can be considered an expected effect size. In Additional file 1: Appendix D of the online supplementary material we conduct a simulation study that shows that the model-based counterfactual values are close to the true unobserved counterfactual values. Moreover, given the current study design, causal inference is not possible. It is impossible to eliminate possible confounders, and the lack of a control group makes causal inference difficult. However, an ITS is a strong quasi-experimental study design [17, 18] and is appropriate given that there is no ethical alternative, as is the case of all-cause mortality for Covid-19. Finally, our results on excess mortality rates in Israel generally replicated among groups with differential mortality risks, and are consistent with previous studies on excess mortality in Israel following the Covid-19 pandemic [15, 16]. It is debatable whether the effect of Covid-19 on all-cause mortality extends to other nations. However, our method and implementation for effect sizes in ITS are not compromised by this limitation. Future study should consider implementing methods to quantify effect sizes for meta-analysis in ITS.

## Conclusions

The limitations of our study are offset by several strengths. Population-based study design, results consistent with level and slope and readily understood outcome for policy makers. Reporting of effect sizes in ITS is desirable because effect sizes are readily understood and capture an entire intervention effect in a single value. The estimator of the effect size is based on the changes in the level and slope of the regression coefficients and so extends the classical approach to ITS models. The current study is the first to offer a framework to report effect sizes in a general ITS and includes an easy-to-use R package to fit an ITS and estimate the effect size. Also, for the first time, we used an ITS design to examine the effect of the Covid-19 pandemic on national population-based all-cause mortality rates, demonstrating that the period exposed to the Covid-19 pandemic was associated with an 11% excess all-cause mortality in Israel.

## Supplementary Information

**Additional file 1: Appendix A. **Quantifying the effect size. **Appendix B.** Data analysis example in R. **Appendix C.** Additional figures for the sensitivity analysis. **Figure S1.** Scatterplot and Regression Fitted Values for Males then Females. **Figure S2.** Scatterplot and Regression Fitted Values for Males Over 60 and Females Over 60. **Figure S3. **Scatterplot and Regression Fitted Values for Different Age Groups. **Figure S4.** Scatterplot and Regression Fitted Values for Short Pre-Intervention Period. **Appendix D.** Simulation Study. **Figure S5.** Boxplot of Mean Squared Error.

## Data Availability

Data are available in a public, open access repository. All data and code use for this article are available in an open access repository (https://github.com/Yael-Travis-Lumer/its2es). This repository includes aggregated death counts by dates retrieved from the Israel Central Bureau of Statistics (CBS) form https://www.cbs.gov.il/he/subjects/Pages/%D7%AA%D7%9E%D7%95%D7%AA%D7%94-%D7%95%D7%AA%D7%95%D7%97%D7%9C%D7%AA-%D7%97%D7%99%D7%99%D7%9D.aspx. The data are open to the public and passed CBS approval for access so that individual people cannot be identified. Hence the data and code are accessible for public use.

## Abbreviations

ITS: Interrupted time series
RR: Relative risk
CI: Confidence interval
CBS: Central Bureau of Statistics
