# Supplementary material for: Effect size quantification for interrupted time series analysis: implementation in R and analysis for Covid-19 research
Source: Emerg Themes Epidemiol. 2022 Nov 11;19:9. doi: 10.1186/s12982-022-00118-7 (PMC9652048; doi:10.1186/s12982-022-00118-7)
Supplement: Supplementary file 1 — Additional file 1: Appendix A. Quantifying the effect size. Appendix B. Data analysis example in R. Appendix C. Additional figures for the sensitivity analysis. Figure S1. Scatterplot and Regression Fitted Values for Males then Females. Figure S2. Scatterplot and Regression Fitted Values for Males Over 60 and Females Over 60. Figure S3. Scatterplot and Regression Fitted Values for Different Age Groups. Figure S4. Scatterplot and Regression Fitted Values for Short Pre-Intervention Period. Appendix D. Simulation Study. Figure S5. Boxplot of Mean Squared Error. [file 12982_2022_118_MOESM1_ESM.docx]

Supplement to Effect size quantification for interrupted time series analysis: Implementation in R and analysis for Covid-19 research

Submitted to Emerging Themes in Epidemiology

Contents

[Appendix A – quantifying the effect size 2](#_Toc117580592)

[**Continuous outcomes** 2](#_Toc117580593)

[**Count outcomes** 4](#_Toc117580594)

[Appendix B – Data analysis example in R 7](#_Toc117580595)

[**Load library and data** 7](#_Toc117580596)

[**Fit an ITS Poisson regression model with seasonal adjustments to count outcomes** 7](#_Toc117580597)

[**Define formula** 7](#_Toc117580598)

[**Fit an ITS Poisson regression model to count outcomes** 8](#_Toc117580599)

[**Plot predicted values (fitted values and counterfactual values)** 9](#_Toc117580600)

[Appendix C – Additional figures for the sensitivity analysis 11](#_Toc117580601)

[**eFigure 1 Scatterplot and Regression Fitted Values for Males then Females** 11](#_Toc117580602)

[**eFigure 2 Scatterplot and Regression Fitted Values for Males Over 60 and Females Over 60** 12](#_Toc117580603)

[**eFigure 3 Scatterplot and Regression Fitted Values for Different Age Groups** 13](#_Toc117580604)

[**eFigure 4 Scatterplot and Regression Fitted Values for Short Pre-Intervention Period** 14](#_Toc117580605)

[Appendix D – Simulation Study 15](#_Toc117580606)

[**eFigure 5 Boxplot of Mean Squared Error** 16](#_Toc117580607)

[References 17](#_Toc117580608)

# Appendix A – quantifying the effect size

Here we aim to quantify the mean effect size, where for continuous outcomes we average standardized differences, and for count outcomes we average risk ratios.

## **Continuous outcomes**

Assume the regression model

| $E\left( Y_{t}\mid X_{t},t \right)=\beta_{0}+\beta_{1}\cdot t+\beta_{2}\cdot X_{t}+\beta_{3}\cdot(t-t^{*})X_{t},$ | *(1)* |
| --- | --- |

which corresponds to continuous outcomes. Note that the model before the intervention is

| $E\left( Y_{t}\mid X_{t},t \right)=\beta_{0}+\beta_{1}\cdot t, \forall t=1,\ldots,t^{*}-1,$ | *(*$SEQ Equation \backslash* ARABIC$ $2$*)* |
| --- | --- |

and the model after the intervention is

| $E\left( Y_{t}\mid X_{t},t \right)=\beta_{0}+\beta_{1}\cdot t+\beta_{2}+\beta_{3}\cdot(t-t^{*}), \forall t=t^{*},\ldots,T.$ | *(*$SEQ Equation \backslash* ARABIC$ $3$*)* |
| --- | --- |

The estimated regression coefficients are $\hat{\beta_{j}}$, $0\leq j\leq3$, and the model predictions for time $t$ are obtained by

$$\hat{Y_{t}}=\hat{\beta_{0}}+\hat{\beta_{1}}\cdot t+\hat{\beta_{2}}\cdot X_{t}+\hat{\beta_{3}}\cdot(t-t^{*})X_{t}.$$

Specifically, for a time point $t$ during the intervention period $\{t^{*},\ldots,T\}$ we obtain the prediction

| $\hat{Y_{t}}=\hat{\beta_{0}}+\hat{\beta_{1}}\cdot t+\hat{\beta_{2}}+\hat{\beta_{3}}\cdot(t-t^{*}),$ | *(*$SEQ Equation \backslash* ARABIC$ $4$*)* |
| --- | --- |

corresponding to ($3$). The counterfactual expected value for time $t\in\{t^{*},\ldots,T\}$, corresponding to the prediction had the intervention not occurred, can be obtained similarly using ($2$):

| $\hat{Y_{t}^{C}}=\hat{\beta_{0}}+\hat{\beta_{1}}\cdot t.$ | *(*$SEQ Equation \backslash* ARABIC$ $5$*)* |
| --- | --- |

Although we do not observe the true counterfactual value, it is possible to capitalize on model ($2$) to obtain the expected counterfactual value, had the intervention not occurred.

Our objective is to estimate the effect size. To attain this objective, we subtract the counterfactual predictions ($5$) from the full predictions ($4$). That is, for each of the intervention time points $t^{*}\leq t\leq T$ we define the difference in predictions by

$$\text{difference}\left( t \right)=\hat{\beta_{0}}+\hat{\beta_{1}}\cdot t+\hat{\beta_{2}}+\hat{\beta_{3}}\cdot(t-t^{*})-\left( \hat{\beta_{0}}+\hat{\beta_{1}}\cdot t \right)=\hat{\beta_{2}}+\hat{\beta_{3}}\cdot(t-t^{*}).$$

Then the overall estimated mean difference $\hat{MD}$ for the intervention period is

$$\hat{MD}=\frac{1}{T-t^{*}+1}\sum_{t=t^{*}}^{T} \text{difference}\left( t \right)=\frac{1}{T-t^{*}+1}\sum_{t=t^{*}}^{T} \hat{\beta_{2}}+\hat{\beta_{3}}\cdot(t-t^{*})=\hat{\beta_{2}}+\hat{\beta_{3}}\cdot\left( \frac{T-t^{*}}{2} \right).$$

Finally, we divide the overall mean difference with the pooled standard deviation of the predictions. That is,

$$\hat{d}=\frac{\hat{\beta_{2}}+\hat{\beta_{3}}\cdot\left( \frac{T-t^{*}}{2} \right)}{S_{p}},$$

where $\hat{\beta_{2}}$ and $\hat{\beta_{3}}$ are the estimated regression coefficients of ($1$), and $S_{p}$ is the pooled standard deviation defined by $S_{p}=\sqrt{\frac{S_{1}^{2}+S_{2}^{2}}{2},}$ and where $S_{1}^{2}$ and $S_{2}^{2}$ are the estimated variances of the fitted values and the predicted counterfactual values, respectively. That is, $S_{1}^{2}=\frac{1}{T-t^{*}}{\sum_{t=t^{*}}^{T} \hat{{(Y}_{t}}-\hat{\mu})}^{2}$ and $S_{2}^{2}=\frac{1}{T-t^{*}}{\sum_{t=t^{*}}^{T} \hat{{(Y}_{t}^{C}}- \hat{\mu^{C}})}^{2}$, where $\hat{\mu}=\frac{1}{T-t^{*}+1}\sum_{t=t^{*}}^{T} \hat{Y_{t}}$, and $\hat{\mu^{C}}=\frac{1}{T-t^{*}+1}\sum_{t=t^{*}}^{T} \hat{Y_{t}^{C}}$. Finally, the 95% CI and the corresponding p-value can be obtained based on the parametric bootstrap, that is implemented in the its2es R package. Specifically, for the 95% CI we sample 2000 samples from

$$\beta\sim MVN(\hat{\beta}, \hat{cov(\beta))},$$

where $\hat{\beta}=\left( \hat{\beta_{0}},\hat{\beta_{1}},\hat{\beta_{2}},\hat{\beta_{3}} \right)^{T}$. For each such sample we generate the associated fitted values ($4$) and counterfactual values ($5$) and then quantify the associated Cohen’s *d* of the bootstrap sample, denoted by $\hat{d}_{i}$, $1\leq i\leq2000$. Finally, we take the 2.5% and 97.5% quantile of all the bootstrap sampled $\hat{d}_{i}$’s to obtain a 95% CI. Similarly, to obtain the *P*-value we repeat the same process under the null hypothesis that $\beta_{2}=\beta_{3}=0$, with

$$\beta\sim MVN(\hat{\beta^{N}} , \hat{cov(\beta))},$$

where $\hat{\beta^{N}}=\left( \hat{\beta_{0}},\hat{\beta_{1}},0,0 \right)^{T}$. We then obtain 2000 estimates of Cohen’s *d* under the null hypotheses, denoted by  $\hat{d}_{i}^{N}$. The P-value can now be obtained by *P*$=\frac{1}{2000}\sum_{i=1}^{2000} 1_{\{\left| \hat{d}_{i}^{N} \right|>\left| \hat{d} \right|\}} .$

**Count outcomes**

Assume that we observe a time series of count outcomes $Y_{t}$. An interrupted time series (ITS) Poisson regression model corresponding to both a level change and a slope change following the intervention, can be described as follows:

| $\text{log}E\left( Y_{t}\mid t,X_{t} \right)=\beta_{0}+\beta_{1}t+\beta_{2}X_{t}+\beta_{3}(t-t^{*})\cdot X_{t},$ | *(*$SEQ Equation \backslash* ARABIC$ $7$*)* |
| --- | --- |

where $\beta_{0}$ and $\beta_{1}$ are the pre-intervention initial level and time trend coefficient, respectively; and $\beta_{2}$ and $\beta_{3}$ are the post-intervention level change and slope change, respectively. As before, the model ($7$) can be divided into two parts, before and after the intervention. The model before the intervention is

| $\text{log}E\left( Y_{t}\mid X_{t},t \right)=\beta_{0}+\beta_{1}\cdot t, \forall t=1,\ldots,t^{*}-1,$ | *(*$SEQ Equation \backslash* ARABIC$ $8$*)* |
| --- | --- |

and the model after the intervention is

| $\text{log}E\left( Y_{t}\mid X_{t},t \right)=\beta_{0}+\beta_{1}\cdot t+\beta_{2}+\beta_{3}\cdot\left( t-t^{*} \right), \forall t=t^{*},\ldots,T.$ | *(*$SEQ Equation \backslash* ARABIC$ $9$*)* |
| --- | --- |

The model predictions for the intervention period are

| $\hat{Y_{t}}=\text{exp}\left( \hat{\beta_{0}}+\hat{\beta_{1}}\cdot t+\hat{\beta_{2}}+\hat{\beta_{3}}\cdot(t-t^{*}) \right), \forall t\in\{t^{*},\ldots,T\}$ | *(*$SEQ Equation \backslash* ARABIC$ $10$*)* |
| --- | --- |

corresponding to ($9$). The counterfactual expected value for time $t\in\{t^{*},\ldots,T\}$, corresponding to the prediction had the intervention not occurred, can be obtained similarly using ($8$):

| $\hat{Y_{t}^{C}}=\text{exp}\left( \hat{\beta_{0}}+\hat{\beta_{1}}\cdot t \right).$ | *(*$SEQ Equation \backslash* ARABIC$ $11$*)* |
| --- | --- |

Additional generalizations of the Poisson model, such as adjustment to over-dispersion, modeling rate data, or adjusting for seasonality, are implemented in the its2es R package.

To estimate the effect size in an ITS with count data, we offer a formula to quantify the relative risk (RR). First, we calculate the point-wise RR for each of the time points in the intervention period, and then use a geometric mean to obtain the final RR for the entire intervention period, as we are averaging rates. We estimate the RR at time $t$, $t^{*}\leq t\leq T$ by

$$\text{RR}\left( t \right)=\frac{\text{exp}\left( \hat{\beta_{0}}+\hat{\beta_{1}}\cdot t+\hat{\beta_{2}}+\hat{\beta_{3}}\cdot(t-t^{*}) \right)}{\text{exp}\left( \hat{\beta_{0}}+\hat{\beta_{1}}\cdot t \right)}=\text{exp}\left( \hat{\beta_{2}}+\hat{\beta_{3}}\cdot(t-t^{*}) \right),$$

where the predictions ($10$) are the numerator, and the predictions ($11$) are the denominator. The final RR estimate for the entire intervention period can now be calculated using a geometric mean

$$\text{RR}=\left( \prod_{t=t^{*}}^{T} \text{RR}\left( t \right) \right)^{\frac{1}{T-t^{*}+1}}=\text{exp}\left( \hat{\beta_{2}}+\frac{T-t^{*}}{2}\cdot\hat{\beta_{3}} \right)=\text{exp}\left( \hat{MD} \right),$$

where the equality before last follows from exponentiation rules. A 95% confidence interval for the RR is given by

$$\text{CI}=\left[ \text{exp}\left( \hat{MD}-1.96\cdot\hat{\sigma}_{MD} \right), \text{exp}\left( \hat{MD}+1.96\cdot\hat{\sigma}_{MD} \right) \right],$$

where 1.96 is the 97.5% percentile point of the standard normal distribution, and where $\hat{\sigma}_{MD}$ is defined by

$\hat{\sigma}_{MD}=\sqrt{var\left( \hat{\beta_{2}} \right)+\left( \frac{T-t^{*}}{2} \right)^{2}\cdot var\left( \hat{\beta_{3}} \right)+2\cdot\frac{T-t^{*}}{2}\cdot cov\left( \hat{\beta_{2}},\hat{\beta_{3}} \right)}$.

The *P*-value for the RR can be calculated by $P=2\left( 1-\Phi\left( \left| \frac{\hat{MD}}{\hat{\sigma}_{MD}} \right| \right) \right)$, where $\Phi\left( \cdot\right)$ is the cumulative distribution function of a standard normal random variable.

Note that the specific case of count outcomes and an impact model assuming no post-intervention slope change (i.e. $\beta_{3}=0$), we obtain the same RR presented in (1–3).

In addition, when seasonality is also considered, the RR is also estimated by $\text{exp}\left( \hat{MD} \right)$ as the seasonal terms cancel out during the division. That is, our measure is robust to the addition of seasonal terms.

# Appendix B – Data analysis example in R

## **Load library and data**

First we load the `its2es’ package which contains the Israeli all-cause mortality data. This is the same data-set analyzed in the current study.

library(its2es)
data <- its2es::Israel_mortality

## **Fit an ITS Poisson regression model with seasonal adjustments to count outcomes**

Here we show how to fit an ITS Poisson regression model to the number of deaths. This is the same model and data used in the primary analysis of our paper.

### **Define formula**

We need to define both a formula object, and the intervention start index. The minimal formula must include the response (count outcome) on the left-hand side of the ~ operator, and the time covariate (a monthly sequence ranging between 1 for January 2001 and 245 for May 2021) on the right. Any additional covariates can also be passed to the right-hand side of the formula, separated by + operators.

form <- as.formula("monthly_total ~ time")

intervention_start_ind <- which(data$Year==2020 & data$Month>2| data$Year==2021)[1]

Note that when the relationship between the time covariate and the outcome is non-linear, one may add flexible spline functions to the ITS regression model to account for any non-linear long-term time trends (1). This can be implemented in the its2es R package as additional covariates to the ITS regression model, using the following modification to the code:

library(splines)

spl <- data.frame(bs(data$time, df=3))

data <- cbind(data,spl)

form <- as.formula(paste("monthly_total ~ ",paste(colnames(spl), collapse= "+")))

### This modification is not modeled and fitted in this example, because this dataset exhibits a linear relationship between the all-cause mortality rate and time (with seasonal fluctuations).

### **Fit an ITS Poisson regression model to count outcomes**

Next, we need to call the its_poisson() function to fit the ITS regression model and to quantify the effect size. We add the total population as our offset term (as we are interested in the mortality rate), and we set the overdispersion argument to TRUE (as the data is over-dispersed) and hence a quasi-Poisson regression model will be used. We use a frequency of 12, corresponding to monthly data, Fourier seasonal adjustments, and a full impact model including both a level change and a slope change following the intervention. Additionally, we set the counterfactual argument to TRUE as we are interested in plotting both the fitted values, and the model-based counterfactual values. For illustration purposes, we print the full model summary by setting the print_summary argument to TRUE.

fit <- its_poisson(data=data,
 form=form,
 offset_name = "monthly_est",
 time_name = "time",
 intervention_start_ind=intervention_start_ind,
 over_dispersion=TRUE,
 freq=12,seasonality= "full",
 impact_model = "full",
 counterfactual = TRUE,
 print_summary = TRUE)

##
## Call:
## glm(formula = form_update_full, family = quasipoisson, data = data)
##
## Deviance Residuals:
## Min 1Q Median 3Q Max
## -13.742 -5.382 -2.520 5.977 20.314
##
## Coefficients:
## Estimate Std. Error t value Pr(>|t|)
## (Intercept) -7.6459355 0.0160954 -475.040 < 2e-16 ***
## time -0.0006608 0.0001176 -5.621 5.47e-08 ***
## indicator 0.0805171 0.0559328 1.440 0.151359
## S1.12 0.0729334 0.0106989 6.817 8.09e-11 ***
## C1.12 0.0974188 0.0107439 9.067 < 2e-16 ***
## S2.12 0.0388667 0.0106732 3.642 0.000335 ***
## C2.12 0.0048053 0.0107091 0.449 0.654060
## S3.12 0.0104348 0.0106555 0.979 0.328468
## C3.12 0.0171351 0.0107271 1.597 0.111558
## S4.12 0.0235026 0.0106662 2.203 0.028554 *
## C4.12 0.0017493 0.0107153 0.163 0.870461
## S5.12 0.0264288 0.0106700 2.477 0.013972 *
## C5.12 0.0066324 0.0107053 0.620 0.536170
## C6.12 -0.0043803 0.0075559 -0.580 0.562668
## indicator:shifted_time 0.0028379 0.0064168 0.442 0.658709
## ---
## Signif. codes: 0 '***' 0.001 '**' 0.01 '*' 0.05 '.' 0.1 ' ' 1
##
## (Dispersion parameter for quasipoisson family taken to be 47.78784)
##
## Null deviance: 20359 on 244 degrees of freedom
## Residual deviance: 10785 on 230 degrees of freedom
## AIC: NA
##
## Number of Fisher Scoring iterations: 4
##
## RR 2.5% CI 97.5% CI P-value
## 1.105594 1.038413 1.177121 0.000000

Finally, note that in cases where the count outcome has an excess number of zeros, one should use a zero-inflated Poisson model. The zero-inflated Poisson model is implemented in the its2es R package, using the function its_zero_inflated_poisson() which is similar in syntax to the function its_poisson(). However, such a modification is not considered in this example, since the mortality data is not zero inflated.

### **Plot predicted values (fitted values and counterfactual values)**

We use the function plot_its_poisson() to plot the predicted values (fitted values and counterfactual values), together with a scatter plot of the outcome. For the first argument, we use the updated data that includes both the fitted values and the model-based counterfactual values. The function requires the intervention start index, the ylabel for the figure, the column name of the count outcome, and the column name of the date column. Note that the additional argument offset_name, which is specific to Poisson regression, can either be set to NULL, in which case the predictions and the observed outcomes are plotted on their original count scale, or set to the column name of the offset term (if it exists), in which case the predictions and the outcome will be divided by the offset and multiplied by 100 to present the percent instead of the count. Below is the code to obtain Fig. 2, where we present the all-cause mortality percent.

p <- plot_its_poisson(data=fit$data,
 intervention_start_ind=intervention_start_ind,
 y_lab="All-cause mortality percent",
 response="monthly_total",
 offset_name = "monthly_est",
 date_name= "Date")

# Appendix C – Additional figures for the sensitivity analysis

## **eFigure 1 Scatterplot and Regression Fitted Values for Males then Females**


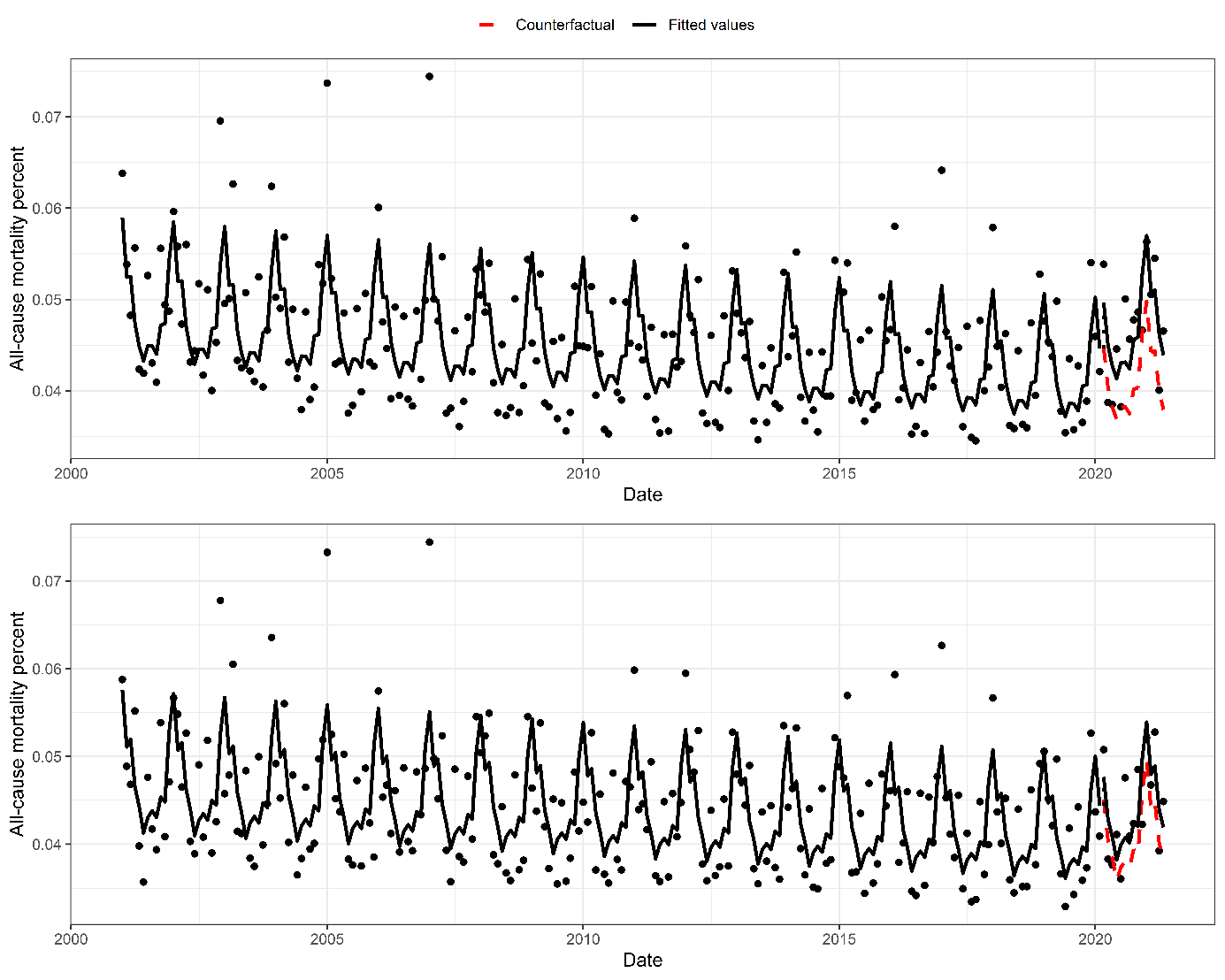


Note. Males top panel, females lower panel. Scatter plot of the monthly mortality percent, together with the regression fitted values (in black), and the counterfactual (in red).

## **eFigure 2 Scatterplot and Regression Fitted Values for Males Over 60 and Females Over 60**


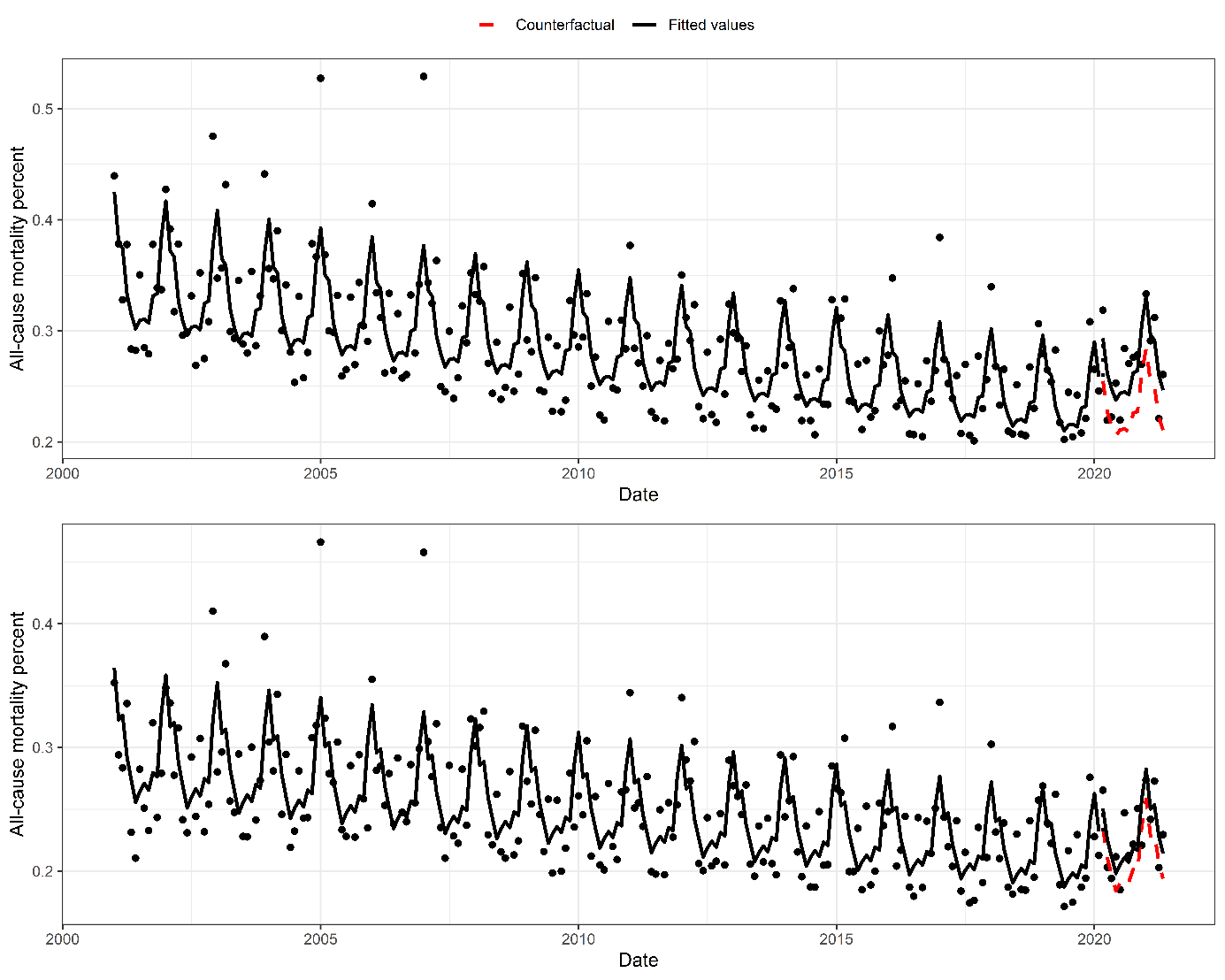


Note. Males over 60 top panel, females over 60 lower panel. Scatter plot of the monthly mortality percent, together with the regression fitted values (in black), and the counterfactual (in red).

## **eFigure 3 Scatterplot and Regression Fitted Values for Different Age Groups**


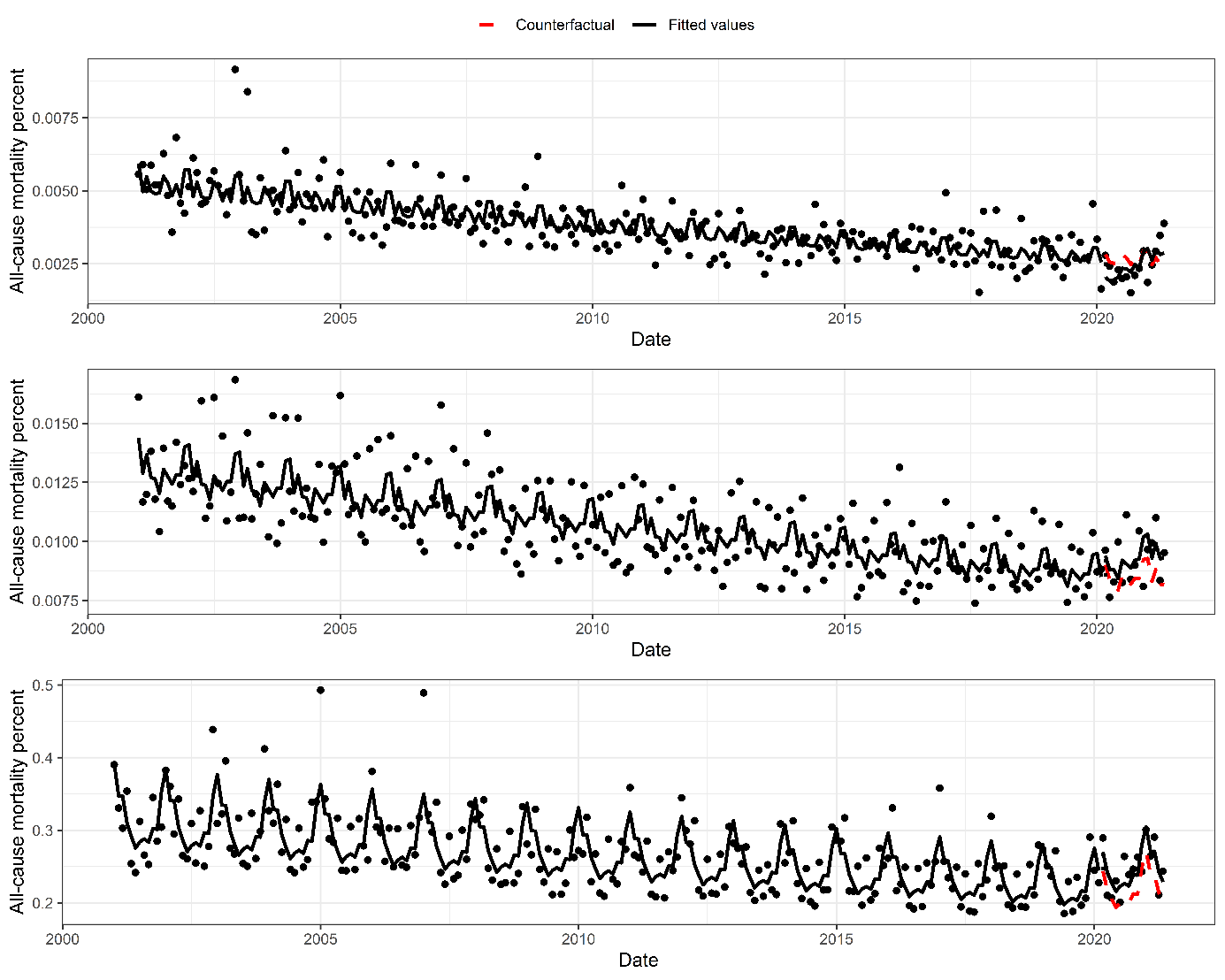


Note. Top ages 0-19, middle ages 20-59, bottom ages over 60. Scatter plot of the monthly mortality percent, together with the regression fitted values (in black), and the counterfactual (in red).

## **eFigure 4 Scatterplot and Regression Fitted Values for Short Pre-Intervention Period**


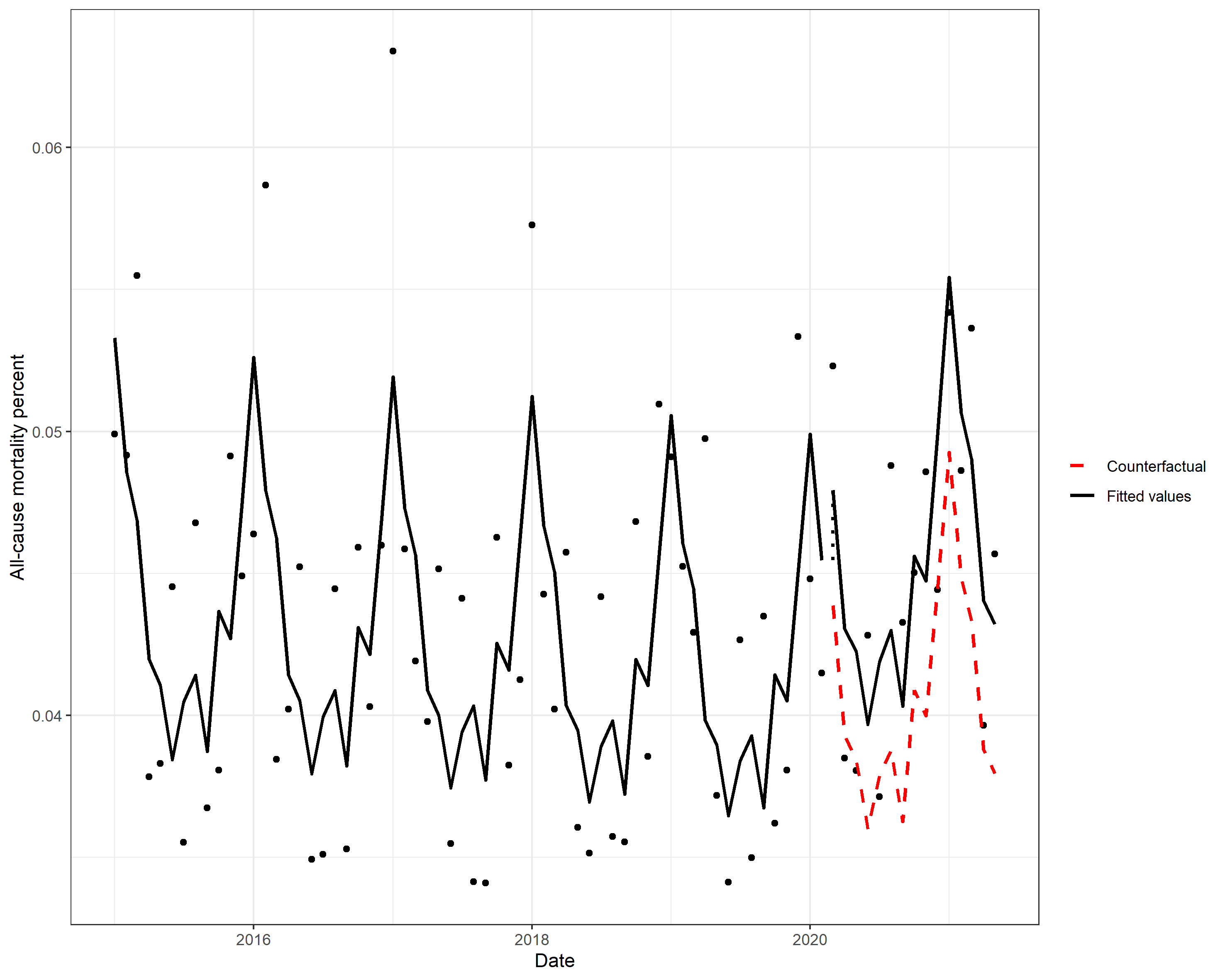


Note. Scatter plot of the monthly mortality percent, together with the regression fitted values (in black), and the counterfactual (in red), for data starting from January 2015.

# Appendix D – Simulation Study

Here we validate our methodology using a simulation study, where we focus on the estimated counterfactual values in the linear regression model (1). We use the following values of regression coefficients: $\beta_{0}=2, \beta_{1}=0.2,\beta_{2}=10, \beta_{3}=0.5.$For each of the intervention time-points, $t\in\{t^{*},\ldots,T\}$, the true (unobserved) counterfactual values are obtained according to $Y_{t}^{true}=\beta_{0}+\beta_{1}\cdot t.$ We then sample from a multi-variate normal (MVN) distribution centered at the true values of the regression coefficients. That is, we sample from $\tilde{\beta}\sim MVN\left( \beta,\hat{\Sigma} \right),$ where $\beta=\left( \beta_{0},\beta_{1},\beta_{2},\beta_{3} \right)^{T}$ is the true (unknown) vector or regression coefficients, and $\hat{\Sigma}$ is the variance of the estimated regression coefficients which we calculated using the Monte Carlo method. For each such sample, we estimate the counterfactual values  $\tilde{Y_{t}}=\tilde{\beta_{0}}+\tilde{\beta_{1}}\cdot t$, and compare them to the true (unobserved) counterfactual values. The mean square error (MSE) between the true unobserved counterfactual values and the estimated counterfactual values is obtained by $\frac{1}{T-t^{*}+1}\sum_{t=t^{*}}^{T} \left( Y_{t}^{true}-\hat{\tilde{Y_{t}}} \right)^{2}$. We repeated these calculations 100 times, each time sampling from different values of $\tilde{\beta}$. eFigure 5 presents the boxplot of the MSEs, based on these 100 simulations. As can be seen, the MSEs are relatively small, indicating that the estimated counterfactual values are close to the true unobserved counterfactual values.

**eFigure 5 Boxplot of Mean Squared Error**


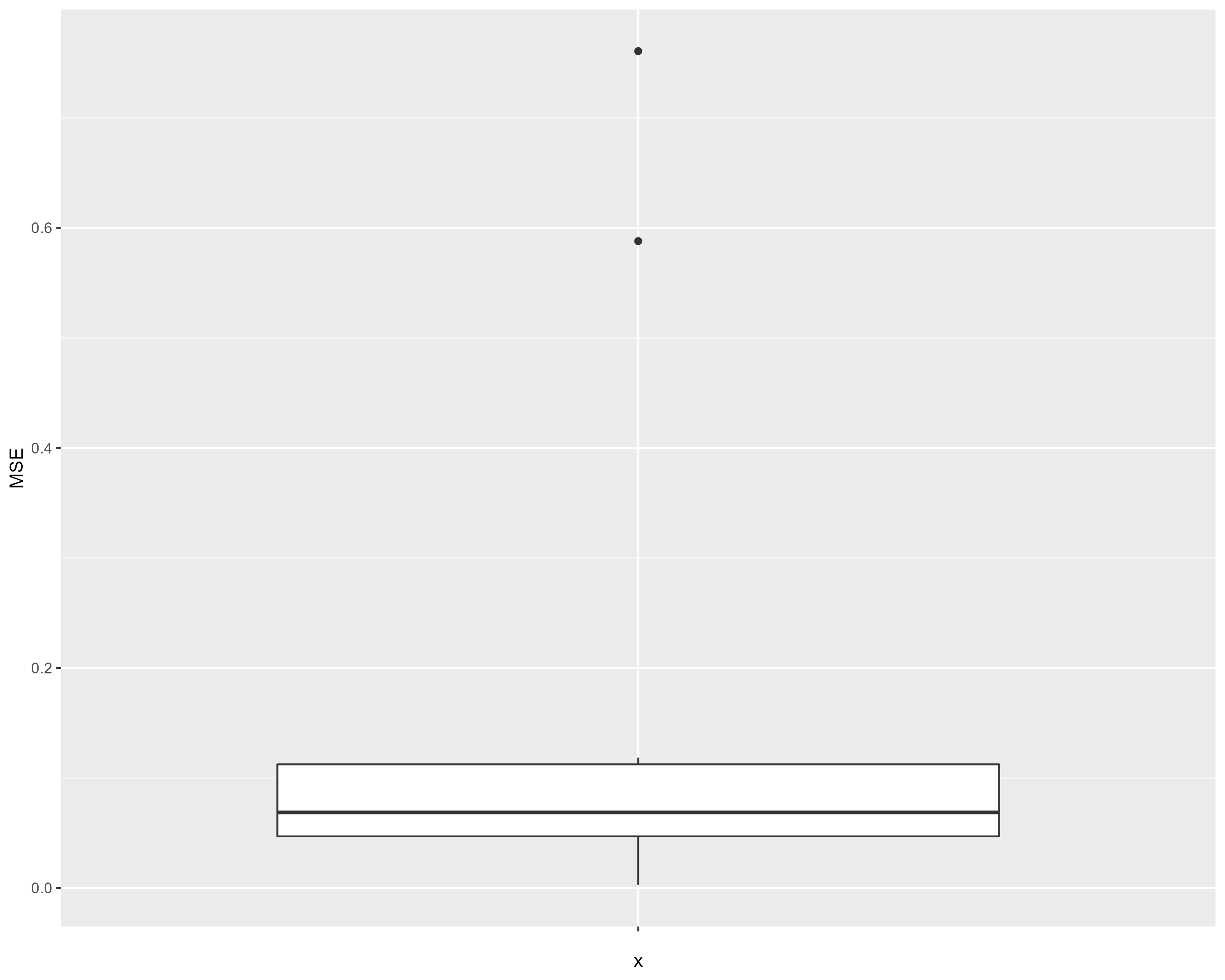


Note. Boxplot of mean squared error (MSE) between the true unobserved counterfactual values, and the estimated counterfactual values, based on 100 samples.

# References

1. Bhaskaran K, Gasparrini A, Hajat S, Smeeth L, Armstrong B. Time series regression studies in environmental epidemiology. Int J Epidemiol. 2013 Aug;42(4):1187–95.

2. Bernal JL, Cummins S, Gasparrini A. Interrupted time series regression for the evaluation of public health interventions: a tutorial. Int J Epidemiol. 2017 Feb 1;46(1):348–55.

3. Bernal JL, Cummins S, Gasparrini A. Corrigendum to: Interrupted time series regression for the evaluation of public health interventions: a tutorial. Int J Epidemiol. 2020 Aug 1;49(4):1414.
